# Multidomain Cognitive Training Transfers to Attentional and Executive Functions in Healthy Older Adults

**DOI:** 10.3389/fnhum.2020.586963

**Published:** 2020-11-16

**Authors:** Patrick D. Gajewski, Sven Thönes, Michael Falkenstein, Edmund Wascher, Stephan Getzmann

**Affiliations:** ^1^Leibniz Research Centre for Working Environment and Human Factors (IfADo), Dortmund, Germany; ^2^Department of Psychology, University of Mainz, Dortmund, Germany; ^3^Institute for Working, Learning and Aging (ALA), Bochum, Germany

**Keywords:** cognitive training, transfer, stroop interference, executive functions, selective attention, verbal fluency

## Abstract

Healthy aging is associated with deficits in focused and sustained attention and executive functions. However, cognitive training (CT) provides a promising method to counteract these deficits. In the present randomized controlled study, we examined to what extent CT regimes can improve attention, verbal skills, and inhibition capacities. Over a period of 16 weeks, healthy older adults (65 years and older, mean: 70 years) received a trainer-guided multidomain paper-and-pencil and computerized CT. Pre- and post-training, a battery of psychometric tests was applied that measured the critical functions. This study used two control groups: a passive control and an active control group performing a relaxation training. Compared to a passive control group, the CT led to enhanced performance in the attentional endurance test and the interference list of the Stroop test, whereas no benefits in verbal and crystalized tests were found. Similar effects were found on the attentional endurance compared to the active control group. Additionally, word fluency was enhanced after CT, but the improvement in the Stroop test did not reach significance compared to the active control. The contents of CT were dissimilar to the psychometric tests showing far transfer, whereas no transfer to attentional or memory functions in the daily life assessed by the Cognitive Failures Questionnaire was found. This demonstrates specific gains of multidomain CT on cognitive functions not explicitly trained and lack of transfer to daily activities.

## Introduction

People in western societies are living longer and longer, and the incidence of mild cognitive impairment (MCI) and dementia has increased rapidly from decade to decade. The expected number of people who will be suffering from dementia is estimated at about 150 million in 2050 (WHO, 2015). But even healthy aging is usually associated with a decline of a number of distinct cognitive abilities which are essential for mobility and independent living. Mnemonic, attentional, and executive functions orchestrating goal-directed behavior are especially affected, which compromises the quality of life in older age (Craik and Salthouse, 2000). Thus, in recent years, there is an increasing interest in the mechanisms and determinants of cognitive aging. On the one hand, there are genetic dispositions and other biological factors that set an individual range of cognitive abilities. On the other hand, there are several environmental factors and behavioral adjustments, like education, nutrition, physical activity, and cognitive engagement, that stimulate the cognitive system and may compensate some deficits to a certain extent due to neuronal plasticity even in older age (Stern, 2009; Greenwood and Parasuraman, 2010; Bamidis et al., 2014; Ballesteros et al., 2014, 2015; Gajewski and Falkenstein, 2016, for reviews). Thus, in addition to the influencing factors, methods and interventions ameliorating cognitive aging has also gained more and more interest.

### Cognitive Training and Transfer

One method for improving cognitive fitness in older age is cognitive training (CT). CT for older adults is increasingly used to improve their performance in cognitive functions which are essential for activities in daily life and to maintain self-sufficiency but prone to age-related decline. In most CT studies, test-like tasks or serous games were trained by applying paper-and-pencil tasks or PC-based tasks for a period of several weeks or months. In particular, computer-based games were frequently used to assess the efficacy of CT. Indeed, a number of controlled studies showed effects of computer-based CT on attention (Green and Bavelier, 2003, 2006; Schubert et al., 2015; Strobach and Karbach, 2016; Bavelier and Green, 2019), executive functions (Strobach et al., 2012; Anguera et al., 2013), and working memory (Salminen et al., 2016; Strobach and Huestegge, 2017).

An important aspect of CT research is the transfer effect referring to the ability to use the knowledge and skills learned in one scenario to achieve different goals in other scenarios (Wenig et al., 2019). Transfer can be differentiated into near-transfer effects (post-training improvement in tasks similar to the training tasks) and far-transfer effects (post-training improvement in tasks that are different from the training tasks in nature or in appearance; Barnett and Ceci, 2002). Recent reviews and meta-analyses suggest that CT leads to improvements of the trained cognitive functions (near transfer) and also transfers to untrained cognitive tasks, intelligence, or even performance in everyday situations (far transfer) in healthy older adults (Baltes et al., 1989; Willis et al., 2006; Ball et al., 2007; Cassavaugh and Kramer, 2009; Karbach and Verhaeghen, 2014; Kelly et al., 2014; Strobach and Karbach, 2016; Chiu et al., 2017; Strobach and Huestegge, 2017).

### Limits of Cognitive Training

The outcome of CT is usually indicated by performance changes in psychometric or neuropsychological tests. As outlined above, studies using behavioral measures showed training-related improvements of mnemonic and executive functions (Kueider et al., 2012; Lampit et al., 2014, for meta-analyses). These cognitive functions have been assumed to constitute fluid intelligence that enables planning and implementing goal-directed behavior, and the flexible coping with new and unexpected situations. In contrast, crystallized intelligence representing general knowledge and life experience as originally proposed by Horn and Cattell (1967) (see also Baltes, 1987) usually remains unchanged after CT (Baltes et al., 1989). Analyses of brain processes accompanied by CT-related gains of cognitive performance have repeatedly shown enhanced activity in EEG- and fMRI-based measures (Bamidis et al., 2014; Brehmer et al., 2014; Falkenstein and Gajewski, 2016). Nevertheless, the findings regarding training efficacy and transfer are inconsistent. Some meta-analyses showed benefits of CT on a number of cognitive functions, such as memory, attention, and executive functions, whereas other meta-analyses did not find substantial effects or found only marginal effects of CT (Lampit et al., 2014; Simons et al., 2016). It seems that the efficacy of CT depends on a large number of influencing factors, such as training content, duration and frequency of CT, adaptivity of the training to cognitive changes, amount of overlap of neural circuits affected by training contents, and performance measure but also type of measures and study design (Bavelier and Green, 2019).

Moreover, a potential shortcoming of CT regimes is a rather inconsistent transfer to other, not explicitly trained cognitive functions. On the one hand, there are several positive findings regarding near or even far transfer (Willis et al., 2006; Minear and Shah, 2008; Karbach and Kray, 2009; Karbach and Verhaeghen, 2014; Strobach and Huestegge, 2017), while some studies and meta-analyses questioned the existence of far transfer on non-trained functions (Shipstead et al., 2012; Melby-Lervåg and Hulme, 2013; Melby-Lervåg et al., 2016; Sala and Gobet, 2017). As a consequence, more randomized controlled studies are necessary to elucidate the efficacy of training and transfer effects of CT on cognitive functions in old age.

### The Present Study

The aim of the present study was to evaluate effects of multidomain adaptive paper-and-pencil and computerized CT in older individuals in a randomized controlled trial. Multidomain CTs are not limited to a single cognitive process (such as memory or processing speed) but involve a number of cognitive processes that interact and increase the demands on the cognitive system (Cheng et al., 2012). Indeed, the new aspect of the present study was the complexity and diversity of the CT that aimed at maximal enhancement of general cognitive functioning in the participants by offering them an interesting and varying program with a large fun factor to enhance their motivation to train. Moreover, the training was adaptive. Adaptivity refers to an adjustment of the training difficulty to an individual performance level and a continuous feedback and has been shown to enhance the motivation of participants and to produce larger transfer effects than non-adaptive trainings (Jaeggi et al., 2008; Karbach and Kray, 2009; Anguera et al., 2013; Mishra et al., 2014). Thus, enhanced complexity, adaptivity, and increasing difficulty of the CT should improve subject’s cognitive capacity and thus increase the probability to achieve transfer effects to non-trained functions.

The data presented here are from a large training study of which some PC-based tasks using electrophysiological measures have already been reported (Gajewski and Falkenstein, 2012, 2018; Wild-Wall et al., 2012; Küper et al., 2017). For the present study, the results of standardized psychometric paper-and-pencil tests were analyzed. In particular, we focused on cognitive domains like processing speed, selective and sustained attention, word fluency, and executive functions like interference processing contributing to fluid intelligence. These tests have been widely used to assess effects of CT and to ensure comparability with other training studies.

We compared the performance of older participants before and after a complex adaptive multidomain CT with the performance of an active control (relaxation training) and no-contact control groups. The no-contact control group was implemented to evaluate test–retest effects (Bavelier and Green, 2019). The rationale to use an active control group was to extract effects that are due to the social component and regular activity during the same time with the same frequency as the CT group. Participants were trained for a period of 4 months, twice per week and 90 min per session. We expected that 4 months of multidomain CT is sufficient to improve cognitive functions constituting fluid intelligence in an older population, whereas no changes are expected with regard to crystallized intelligence. In addition, we took a closer look on the domain-specific improvements after CT and evaluated transfer effects of non-explicit trained tasks on attention-related and executive functions and a potential far transfer to everyday attentional and memory functions assessed by the Cognitive Failures Questionnaire (CFQ).

## Materials and Methods

### Participants

**FIGURE 1:**
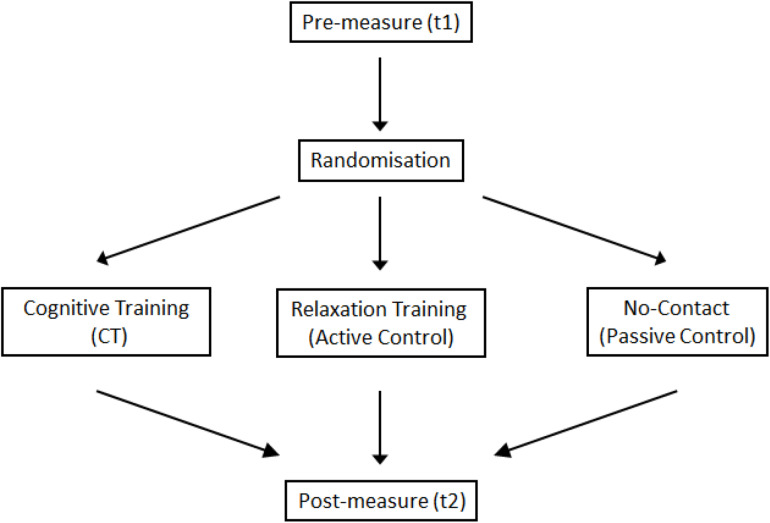
Design of the study.

Data of 69 participants aged 65–88 years (*M* = 70.3 years, *SD* = 4.3, 63.8% female) were analyzed, who conducted either CT, or a relaxation training (active control group), or belonged to a no-contact control group. A fourth group, which was part of the original study that received physical training, was not included here as physical intervention was outside of the focus of the present study. For details on the acquisition procedure and the characteristics of the whole sample, please refer to Gajewski and Falkenstein (2012). Shortly, the participants were included in the study after having met a number of criteria inquired by a telephone interview. They should be physically and mentally fit without any history of neurological, psychiatric, motor, cardiovascular, or oncologic diseases or any psychopharmacological medication. No participants who already engaged in CT for more than 1.5 h/week were included in the study. The participants were randomly assigned to the CT group (CT; *n* = 32, mean age: *M* = 71, *SD* = 4.2, 62.5% female), the active control group (ACG; *n* = 33, mean age: *M* = 71, *SD* = 4.5, 62.9% female), and the passive control group (CG; *n* = 37, mean age: *M* = 70, *SD* = 4.2, 61.5% female). The final number of participants varied between 31 and 32 in the CT group, 33 and 34 in the active control group, and 36 and 37 in the passive control group due to missing data in some of the tests (e.g., due to color blindness or formal errors). The design of the study is illustrated in Figure 1.

The study was carried out in accordance with the Declaration of Helsinki and was approved by the local Ethical Committee of the Leibniz Research Centre for Working Environment and Human Factors, Dortmund, Germany. All participants gave written informed consent and received 100 Euro to recompense them for travel expenses.

### Multidomain Cognitive Training

Participants of the CT group were trained for 16 weeks, twice per week and 90 min per session. The CT was supervised by a professional trainer and a student assistant in small groups of no more than 12 participants. Participants who had missed regular sessions had the opportunity to take part in two additional sessions after the regular training had been completed. In the first step, participants were given basic information on cognitive functions and their relevance for the daily life to enhance compliance and motivation to train. The subsequent multidomain CT consisted of paper-and-pencil tasks and PC-based games. The difficulty of the tasks was adapted and adjusted to the current individual performance of the trainees.

The basic principle of the training was to construct exercises that are as diverse as possible. Therefore, each exercise was performed two times at most, which should ensure that participants find the training interesting and entertaining. It was not intended to train a particular cognitive ability, for example, spatial awareness or reasoning, but to employ different exercises requiring different cognitive abilities. Hence, each participant got the chance to become familiar with different types of training and to find out which ones were most suitable to him or her. Furthermore, every participant was motivated to integrate the training into his or her everyday life after the study was finished. While selecting the training programs, we took care that a low-cost continuation of the training is possible.

During the first 4 weeks, a mental activation training (MAT; Lehrl et al., 1994) that consisted of short paper-and-pencil exercises was used to increase working memory capacity, attention, and speed of information processing during a short span of 10–15 min. Moreover, in the first eight sessions, participants without any computer experience were familiarized with handling a computer. During the following 12 weeks, the participants worked on selected internet-based serious games^1^. See the Appendix for a detailed description of the games and the training schedule. Each session consisted of different games that aimed at training most relevant cognitive functions. The exercises and games mainly involved mnemonic functions, but some of them also included multitasking, logical thinking, cognitive flexibility, spatial reasoning, and aspects of executive functions. However, the elements of these games had no similarity to the psychometric tests used for pre- and post-testing. In particular, the CT consisted of several PC-based games, whereas the cognitive measures reflected well-structured, paper-and-pencil tests without apparent overlapping features with the games. In this case, a transfer effect should be assumed as transfer effects refer to the ability that individuals have to use the knowledge and skills learned in one scenario to achieve different goals in other scenarios. The participants were not encouraged to exercise beyond the training sessions during the study.

### Active and Passive Control Groups

The participants of the active control group were trained similar to the CT group for a period of 4 months, twice per week and 90 min per session. This group received a relaxation training consisting of progressive muscle relaxation, autogenic training, breathing exercises, back training, massage, and Qigong. The aim of this training was to provide interesting and varied exercises which did not involve and hence should not train cognitive functions. The passive control group did not participate in any intervention.

### Testing

Participants completed a number of sociodemographic questionnaires at home. During the test session, a number of paper-and-pencil psychometric tests that assessed a broad spectrum of cognitive abilities like perceptual speed, sustained and focused attention, short and working memory, long-term memory, interference processing, divergent thinking, and verbal abilities were used. After a 1-h break, a second test session using computer-based tasks with EEG recording was conducted, which was not the focus of analysis here (for further details, see Gajewski and Falkenstein, 2012, 2018; Wild-Wall et al., 2012; Küper et al., 2017).

### Psychometric Tests

The focus of interest in the present study was fluid cognitive functions like focused and sustained attention, processing speed, cognitive flexibility, and interference control as one of the crucial executive functions. The selected tests are sensitive to subtle changes in cognitive performance of healthy adults and are mainly used in non-clinical populations. Thus, the tests are even more able to differentiate between persons with normal cognition and those with just the beginnings of MCI. The detailed description of the tests and their psychometric properties are provided in more detail in Gajewski et al. (2018) and are only shortly outlined in the following.

### d2 Test

The d2 test (Brickenkamp, 1972) consists of a sheet presenting 14 lines of 47 letters (d and p), each with one to four dashes (‘), located either individually or in pairs above or below the letter. Participants were asked to go as fast as possible through each line and identify every d with two dashes by crossing it out. After 20 s of processing one line, the subjects were told to move on to the next line and to continue. The total number of correctly identified d’s with two dashes represents the test score. The d2 test is a measure of focused and sustained attention (attentional endurance) as well as processing speed.

### Digit Symbol Test

The digit symbol test (DST) is an evaluation tool used to assess psychomotor functions. Initially, it was part of the Wechsler Adult Intelligence Test (WAIS; Wechsler, 1956). It consists of nine digit–symbol pairs (e.g., 7/Λ) followed by a list of digits. Under each digit, the subject should write down the corresponding symbol as fast as possible. The number of correct symbols produced within the 90 s reflects the test score. The DST measures processing speed, visuospatial processing, and selective attention.

### Word Fluency Test

In the word fluency test (from LPS, Horn, 1983), participants are asked to recall as many words as possible within a given time, each word beginning with a specific letter. Three trials were conducted, in which words with the initial letters B, F, and L were asked for. For the post-measure, a parallel version was used, including the letters P, K, and S. Participants were given 60 s for each trial. The produced words were recorded by the experimenter. The total number of meaningful words represents the test result. The test measures the ability to access the verbal lexicon, semantic memory, scope of vocabulary, cognitive flexibility, and divergent thinking.

### Stroop Test

The Stroop test (from NAI, Oswald and Fleischmann, 1986) consists of three parts. In the first part (Stroop 1), subjects were given a sheet of paper with names of colors printed in black ink. The participants were asked to read them out aloud as fast as possible. In the second part (Stroop 2), participants were handed another sheet of paper with colored bars on it. Participants were asked to name the colors. In the third condition (Stroop 3), subjects were given a sheet of paper with names of colors printed in various colors which did not match the names of the colors (e.g., “GREEN” was printed in red). Subjects had to name the colors the words were printed in as fast as possible and to ignore the meaning of the words. There was the same number of items in every condition. The time needed to perform each condition was analyzed. The time of the third list is considered an indicator of interference processing and inhibitory control as one of the most important executive functions. To assess the baseline-corrected interference, the time of Stroop 2 was subtracted from the time to complete Stroop 3.

### Multiple-Choice Vocabulary Test (MWT-B)

The MWT-B (Lehrl, 1995) assesses crystalline intelligence. The test consists of 37 items with five words each. Only one of the five words reflects a meaningful word; the other similar words are meaningless. The subjects were required to mark the correct word. The difficulty of items increased with the increasing item number. The number of correctly identified meaningful words allows assessment of the crystalline IQ.

### CFQ

To assess a possible far transfer of CT to the performance in daily activities, the CFQ (Broadbent et al., 1982) was used. CFQ is a scale including 26 questions related to attentional and memory lapses in daily life, for example, “Do you find you forget whether you’ve turned off a light or a fire or locked the door?” or “Do you fail to listen to people’s names when you are meeting them?” Frequent lapses are scored higher than less frequent ones. The CFQ was exclusively conducted in the post-test to avoid test–retest effects (cf. Strobach and Huestegge, 2017).

### Statistical Analysis

A series of two-way mixed analyses of variance with repeated measures (mixed ANOVAs) with the factors Group (CT vs. active control group and CT vs. passive control group) and Session (t1 vs. t2) were conducted. Significant interactions were further analyzed using *t*-tests. Mean values with standard errors of the mean are presented (±1 SEM). Estimators of effect size are provided by using partial eta square (η_*p*_^2^). To examine the training gains and transfer effects, we also calculated Cohen’s (1977) *d*_*z*_ or the standardized mean difference in performance between pre- and post-test (Verhaeghen et al., 1992; see also Lakens, 2013). Therefore, the pre-test–post-test differences for the CT and the control groups were divided by the pooled standard deviation for pre- and post-measures for each test. An alternative way to calculate *d*_*z*_ is to divide the *t*-value of the paired-samples *t*-test by the squared number of persons (Lakens, 2013).

## Results

### d2 Test

**FIGURE 2:**
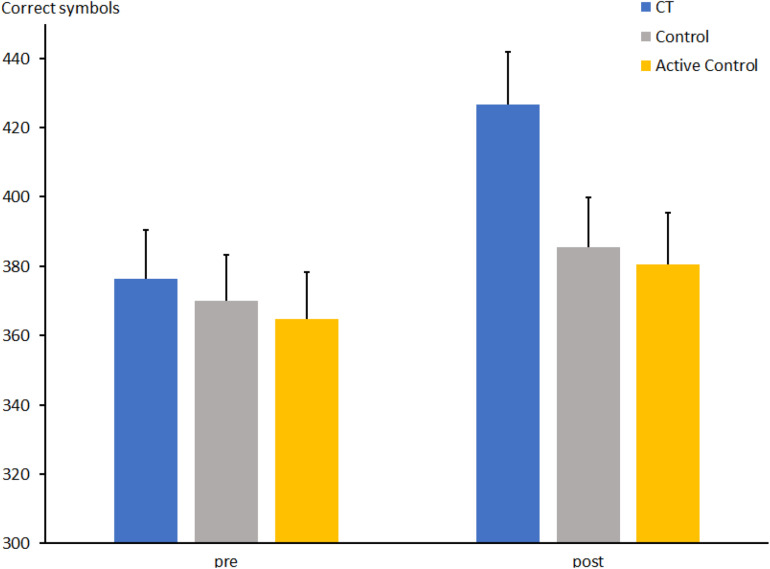
Number of correctly marked symbols in the d2 test at pre- and post-measures in the CT group (CT), passive control group (Control), and active control group (Active Control). Error bars reflect standard errors.

The repeated-measures ANOVA for the comparison between the CT and the passive control groups for the number of correctly crossed d2 symbols (Figure 2) indicated a main effect of Session (t1: 373 ± 9.6 vs. t2: 406 ± 10.5; *F*[1, 66] = 27.0, *p* < 0.0001, η_*p*_^2^ = 0.290) and no effect of Group (*F*[1, 66] = 1.5, *p* = 0.22, η_*p*_^2^ = 0.023). The interaction Group × Session was significant (*F*[1, 66] = 7.4, *p* < 0.01, η_*p*_^2^ = 0.101) and indicated a larger increase of correctly crossed d2s at t2 than at t1 in the CT group [426.3 ± 15.2 vs. 376.3 ± 14.0; *t*(31) = 5.6, *p* < 0.0001] relative to the passive control group [385.5 ± 14.4 vs. 369.9 ± 13.2; *t*(35) = 1.7, *p* = 0.09].

The number of correct symbols in the d2 test for the comparison between the CT and the active control group revealed a main effect of Session, indicating a larger number of crossed symbols at t2 than at t1 (t1: 370 ± 9.5 vs. t2: 403 ± 10.5; *F*[1, 62] = 17.8, *p* < 0.0001, η_*p*_^2^ = 0.223). No main effect of group was found (*F*[1, 62] = 2.4, *p* = 0.12, η_*p*_^2^ = 0.038), but there was an interaction between Group and Session (*F*[1, 62] = 4.8, *p* < 0.05, η_*p*_^2^ = 0.072), indicating a larger increase of the number of symbols at t2 compared to t1 in the CT group as outlined above [426.3 ± 15.2 vs. 376.3 ± 14.0; *t*(31) = 5.6, *p* < 0.0001] than in the active control group [377.9 ± 12.7 vs. 360.7 ± 12.7; *t*(32) = 1.3, *p* = 0.18].

### DST

**FIGURE 3:**
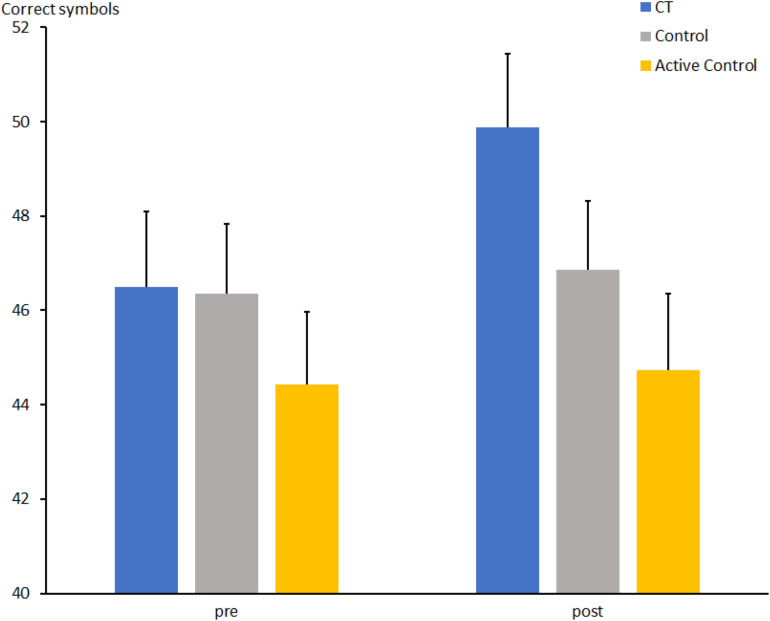
Total number of correctly substituted symbols in the DST at pre- and post-measures in the CT group (CT), passive control group (Control), and active control group (Active Control). Error bars reflect standard errors.

The total number of correctly substituted digit–symbol items for the comparison CT vs. passive control groups (Figure 3) increased from t1 to t2 (t1: 46.4 ± 1.1 vs. t2: 48.0 ± 1.0; *F*[1, 67] = 5.2, *p* < 0.05, η_*p*_^2^ = 0.072). There was no effect of Group (*F*[1, 67] < 1) and a weak trend for an interaction between Group and Session (*F*[1, 67] = 3.2, *p* = 0.079, η_*p*_^2^ = 0.045), indicating a slight increase of correct items from t1 to t2 [46.5 ± 1.6 vs. 49.9 ± 1.6; *t*(31) = 2.7, *p* = 0.011] in the CT group, while no substantial change was observed in the passive control group [46.4 ± 1.5 vs. 46.9 ± 1.5; *t*(36) < 1].

The total number of correctly substituted digit–symbol items for the comparison CT vs. active control increased from t1 to t2 (t1: 45.4 ± 1.0 vs. t2: 47.4 ± 1.1; *F*[1, 63] = 4.4, *p* < 0.05, η_*p*_^2^ = 0.066). There was no main effect of Group (*F*[1, 63] = 2.5, *p* = 0.118, η_*p*_^2^ = 0.038) and again a weak trend for an interaction between Group and Session (*F*[1, 63] = 2.9, *p* = 0.093, η_*p*_^2^ = 0.044), suggesting a slight increase of correct items from t1 to t2 [46.5 ± 1.6 vs. 49.9 ± 1.6; *t*(31) = 2.7, *p* = 0.011] in the CT group, while no substantial change was observed in the active control group [44.4 ± 1.5 vs. 44.7 ± 1.6; *t*(34) < 1].

### Word Fluency Test

**FIGURE 4:**
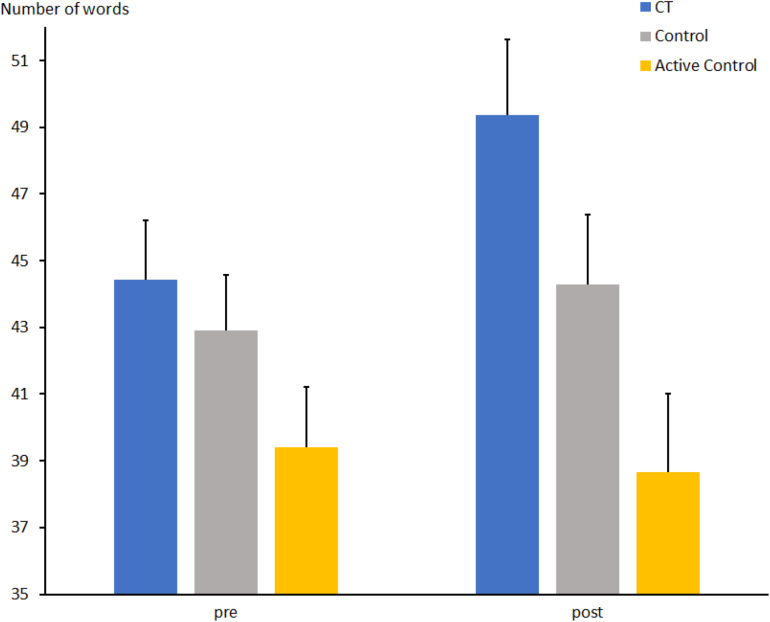
Total number of correctly produced words in the word fluency task at pre- and post-measures in the CT group (CT), passive control group (Control), and active control group (Active Control). Error bars reflect standard errors.

The total number of words for the comparison between CT and passive control groups (Figure 4) increased from t1 to t2 (t1: 43.6 ± 1.2 vs. t2: 46.8 ± 1.5; *F*[1, 67] = 7.2, *p* < 0.01, η_*p*_^2^ = 0.097). There was no effect of Group (*F*[1, 67] = 1.7, *p* = 0.19, η_*p*_^2^ = 0.025). Descriptively, the mean number of words produced by participants of the CT group increased from t1 to t2 [44.4 ± 1.7 vs. 49.4 ± 2.2; *t*(31) = 2.6, *p* = 0.015], while the control group showed only a small increase [43.0 ± 1.6 vs. 44.3 ± 2.1; *t*(36) < 1]. However, the interaction Group × Session did not reach significance (*F*[1, 67] = 2.3, *p* = 0.13, η_*p*_^2^ = 0.033).

The number of words for the comparison between CT and active control groups increased only slightly from t1 to t2 (t1: 41.9 ± 1.3 vs. t2: 44.0 ± 1.6; *F*[1, 63] = 3.0, *p* = 0.085, η_*p*_^2^ = 0.046). In contrast to the comparison above, there was an effect of Group (*F*[1, 63] = 8.2, *p* < 0.01, η_*p*_^2^ = 0.115), due to the generally larger number of words in the CT compared to the active control group (46.9 ± 1.9 vs. 39.9 ± 1.9). Moreover, there was an interaction between Group and Session (*F*[1, 63] = 5.5, *p* < 0.05, η_*p*_^2^ = 0.081), suggesting that the mean number of words produced by participants of the CT group increased from t1 to t2 [44.4 ± 1.7 vs. 49.4 ± 2.2; *t*(31) = 2.6, *p* = 0.015], while the active control group showed no effect [39.4 ± 1.8 vs. 38.6 ± 2.3; *t*(34) < 1].

### Stroop Test

In the first step, a series of ANOVAs were conducted for the CT vs. passive control group and CT vs. active control group for parts 1, 2, and 3 of the Stroop test.

**FIGURE 5:**
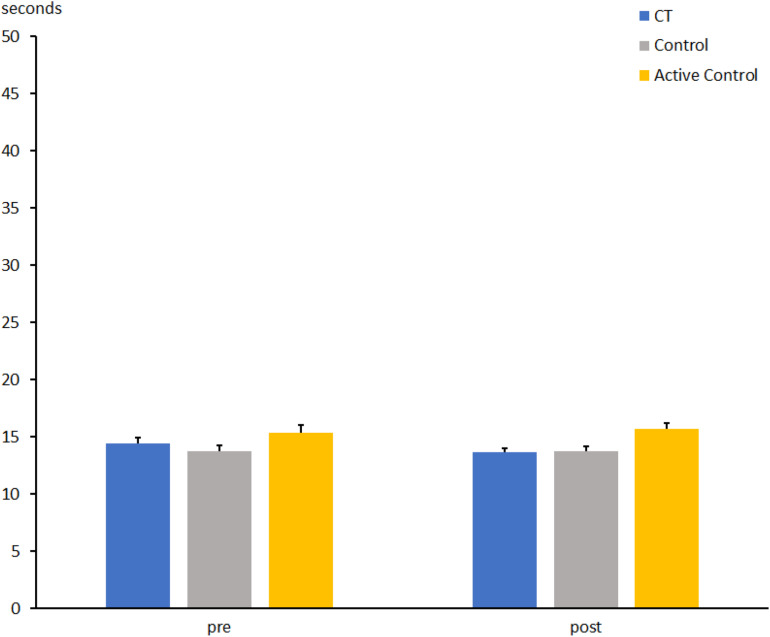
Time to perform part 1 of the Stroop task (reading of color words) at pre- and post-measures in the CT group (CT), passive control group (Control), and active control group (Active Control). Error bars reflect standard errors.

In part 1 of the Stroop test (Figure 5), requiring reading of color words in black ink, the comparison of CT and passive control groups did not show effects of Session (t1: 14.1 ± 0.3 s, t2: 13.7 ± 0.3 s; *F*[1, 65] = 1.8, *p* = 0.18, η_*p*_^2^ = 0.027) or Group (*F*[1, 65] < 1) or an interaction between Group and Session (*F*[1, 65] = 1.9, *p* = 0.17, η_*p*_^2^ = 0.025).

For the comparison between the CT and active control groups in part 1, no effect of Session was observed (t1: 14.8 ± 0.4 s, t2: 14.6 ± 0.3 s; *F*[1, 64] < 1, η_*p*_^2^ = 0.006). However, there was an effect of Group due to faster responses in the CT than in the active control group (14.0 ± 0.5 s, t2: 15.2 ± 0.5 s; *F*[1, 64] = 4.25, *p* < 0.05, η_*p*_^2^ = 0.063). The interaction Group × Session (*F*[1,64] = 2.3, *p* = 0.13, η_*p*_^2^ = 0.035) did not reach significance. However, the main effect of Group (*F*[1, 64] = 4.2, *p* < 0.05, η_*p*_^2^ = 0.062) yielded slower responses in the active control than in the CT group (15.5 ± 0.5 vs. 14.0 ± 0.5 s).

**FIGURE 6:**
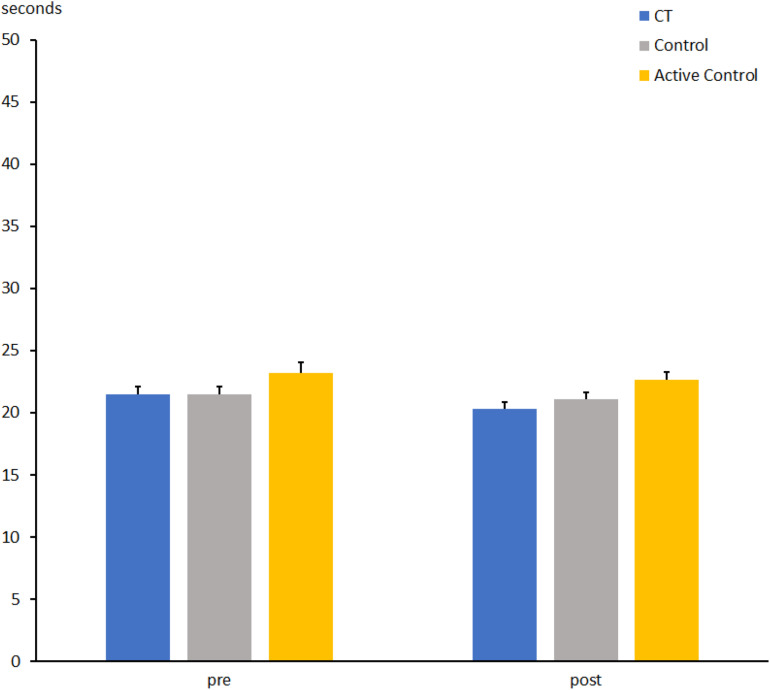
Time to perform part 2 of the Stroop task (naming of color squares) at pre- and post-measures in the CT group (CT), passive control group (Control), and active control group (Active Control). Error bars reflect standard errors.

The time to name color blocks in part 2 of the Stroop test for the CT and passive control groups (Figure 6) was significantly shorter at t2 than at t1 (20.6 ± 0.4 vs. 21.5 ± 0.4; *F*[1, 65] = 6.7, *p* = 0.012, η_*p*_^2^ = 0.094). No Group effect (*F*[1, 65] < 1) or interaction between Group and Session was found (*F*[1, 65] = 1.3, *p* = 0.25, η_*p*_^2^ = 0.020).

For the CT and active control groups, the time to name color blocks in part 2 of the Stroop test was shorter at t2 than at t1 (21.4 ± 0.4 vs. 22.3 ± 0.6; *F*[1, 63] = 4.5, *p* < 0.05, η_*p*_^2^ = 0.066). No interaction between Group and Session was found (*F*[1, 63] < 1, η_*p*_^2^ = 0.008), but again, a main effect of Group was found (*F*[1, 63] = 4.8, *p* < 0.05, η_*p*_^2^ = 0.07), indicating faster responses in CT than in the active control group (CT: 20.8 ± 0.7 vs. 22.9 ± 0.6 s).

**FIGURE 7:**
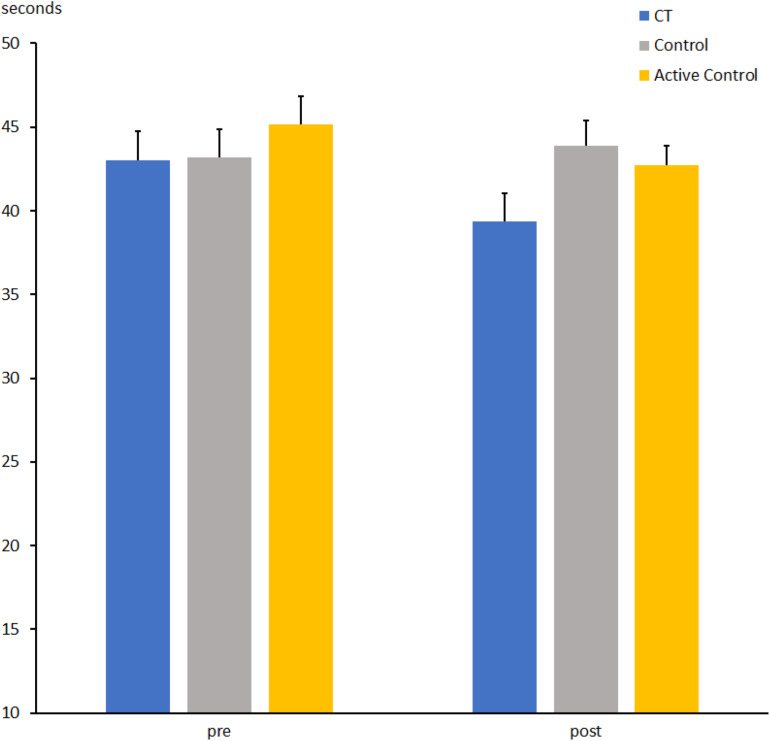
Time to perform part 3 of the Stroop task (interference list) at pre- and post-measures in the CT group (CT), passive control group (Control), and active control group (Active Control). Error bars reflect standard errors.

For the CT and the passive control groups, the time for processing the interference list in part 3 (Figure 7) consisting of incongruent color words was similar at t2 and t1 (41.6 ± 1.2 vs. 43.1 ± 1.2 s; *F*[1, 65] = 2.6, *p* = 0.13, η_*p*_^2^ = 0.038). No Group effect was found (*F*[1, 65] = 1.2, *p* = 0.28, η_*p*_^2^ = 0.018). However, the interaction Group × Session was significant (*F*[1, 65] = 5.4, *p* = 0.023, η_*p*_^2^ = 0.077), indicating faster performance at t2 compared to t1 in the CT group [39.3 ± 1.7 vs. 43.5 ± 1.6; *t*(31) = 2.4, *p* = 0.022], but no substantial difference was detected in the passive control group [43.8 ± 1.5 vs. 43.2 ± 1.6 s; *t*(35) < 1].

The time for processing the interference list in part 3 for the CT and active control groups was shorter at t2 than at t1 (41.0 ± 0.8 vs. 44.0 ± 1.2 s; *F*[1, 63] = 9.5, *p* = 0.005, η_*p*_^2^ = 0.129). No Group effect (*F*[1, 63] = 2.3, *p* = 0.13, η_*p*_^2^ = 0.035) or interaction between Group and Session was found (*F*[1, 63] < 1, η_*p*_^2^ = 0.006).

**FIGURE 8:**
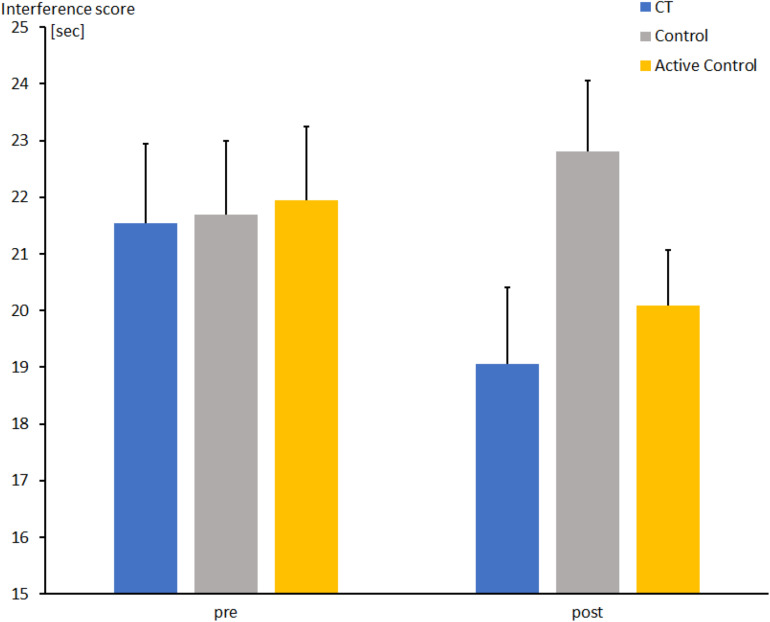
Interference score in seconds (Stroop 3–Stroop 2) at pre- and post-measures in the CT group (CT), passive control group (Control), and active control group (Active Control). Error bars reflect standard errors.

Finally, we analyzed the time difference between Stroop 3 and Stroop 2 to assess the baseline-corrected interference effect (see Figure 8). For the comparison of the CT versus passive control groups, no effect of Session (*F*[1, 65] < 1) or Group was obtained (*F*[1, 65] = 1.4, *p* = 0.24, η_*p*_^2^ = 0.021). Yet the interaction Group × Session was corroborated (*F*[1, 65] = 4.1, *p* < 0.05, η_*p*_^2^ = 0.046), indicating a trend for reduction of interference in the CT group between pre- and post-measures [21.5 ± 0.9 vs. 19.0 ± 0.9 s, *t*(31) = 1.8, *p* = 0.076] and a slight increase between pre- and post-measures in the passive control group [21.6 ± 0.9 vs. 22.8 ± 0.9 s; *t*(36) < 1].

For the comparison between CT and active control, no Group effect or interaction between Group and Session was found (both *F*s < 1). Nevertheless, the effect of Session was corroborated (*F*[1, 63] = 6.0, *p* < 0.05, η_*p*_^2^ = 0.085), indicating a substantial reduction of interference in both groups between pre- and post-measures (21.7 ± 0.9 vs. 19.5 ± 0.7 s).

### MWT-B

**FIGURE 9:**
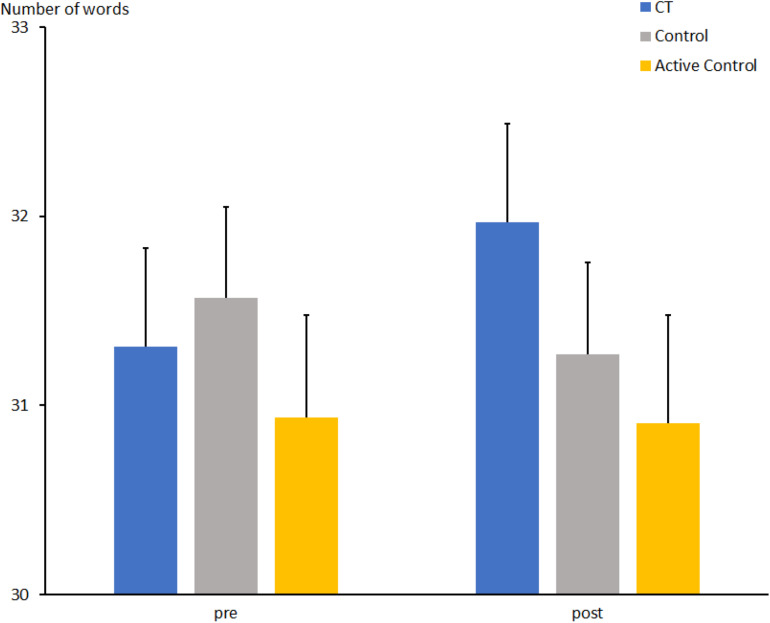
Total number of correctly crossed words in the MWT-B at pre- and post-measures in the CT group (CT), passive control group (Control), and active control group (Active Control). Error bars reflect standard errors.

The number of correctly detected words in the test measuring crystallized intelligence (Figure 9) for the comparison between CT and passive control groups showed no effect of Session or Group (both *F*s[1, 67] < 1) and only a weak trend for the interaction Group × Session (*F*[1, 67] = 3.0, *p* = 0.086, η_*p*_^2^ = 0.043).

Similarly, no effect of Group or Session (both *F*s < 1) or interaction of both factors (*F*[1, 63] = 1.9, *p* = 0.16, η_*p*_^2^ = 0.029) for the comparison between CT and the active control groups was found.

### Training-Related Gains

To assess the training-related gains and transfer effects, Cohen (1977) *d*_*z*_ was calculated by computing pre-test minus post-test differences divided by the pooled standard deviations for pre- and post-tests (Lakens, 2013). A *d*_*z*_ of 1 indicates that the mean difference between pre- and post-test corresponds to one standard deviation. The results for the CT and the control groups are presented in Table 1. As can be seen, the training gains occurred in all of the applied tests after CT relative to the control groups.

**TABLE 1:** Cohens’ *d*_*z*_ reflecting training gains in the CT group (CT) and the passive and active control groups.

Psychometric task	CT	Passive control	Active control
d2 test	1.00	0.29	0.24
Digit symbol test	0.48	0.06	0.08
Word fluency test	0.46	0.15	0.07
Stroop task (part 1)	–0.25	–0.01	–0.11
Stroop task (part 2)	–0.41	–0.19	–0.16
Stroop task (part 3)	–0.43	–0.09	–0.32
Stroop (part 3–part 2)	–0.32	–0.01	–0.27
MWT-B	0.30	0.12	0.05

*Positive values reflect an increased number of correctly performed symbols or words; negative values are due to faster performance in the Stroop task at post- vs. pre-test.*

### Far Transfer to Daily Activities

No group differences in the CFQ were found at the post-test measurement (CT group: 29.2, passive control group: 28.4, active control group: 27.2; both *F*’s < 1).

## Discussion

The present study evaluated effects of a 16-week multidomain CT on different cognitive functions in older participants. Compared to an active control group receiving relaxation training and a passive (no-contact) control group, participants in the CT group showed enhancement of sustained and focused attention (attentional endurance) as assessed by the d2 test. The same pattern was found for the digit symbol substitution test assessing focused attention and psychomotor speed, although the interaction Group × Session showed only a trend compared to both control groups. However, whereas no significant interaction was observed in the word fluency test after CT compared to the passive control group, this difference was substantial in comparison to the active control group. In the simple tasks of the Stroop task (word reading and color naming), no interactions between Group and Session were found (though descriptively, the participants of the CT group showed faster responses than did the control groups). Interestingly, the results of the interference list (Stroop 3), in which interference processing and inhibitory control were required, revealed divergent patterns: while there was a similar improvement of performance in the CT and in the active control groups from pre- to post-measure, the participants of the CT group showed a clear performance improvement after training compared to the passive controls. No effects or interactions were found in the MWT-B that evaluates crystallized intelligence. Thus, crystallized cognitive functions were not improved after training. The findings indicate also that basic cognitive functions like sensory abilities and psychomotor speed were not affected by the CT, whereas higher-order cognitive functions were enhanced. Additionally, though some interactions between Group and Session did not reach significance, the effect sizes of the CT group were consistently larger compared to those of the control groups. For example, the improvement in the d2 task after CT corresponded to one standard deviation. Taken together, these observations indicate that a multidimensional training consisting of a number of short exercises is suitable to improve some attentional and executive functions in older adults. The findings are consistent with a number of previous training studies reporting positive effects of CT on executive functions in older age (Green and Bavelier, 2003; Brehmer et al., 2014; Karbach and Verhaeghen, 2014; Lampit et al., 2014; Ballesteros et al., 2014, 2015; Green et al., 2016).

The comparison between the CT and the two control groups provided similar effects, but there were also some interesting differences in the results. First, word fluency was improved after CT and reached significance relative to the active control group but did not reach significance compared to the passive control group. In contrast, performance in the interference list of the Stroop test was significantly faster in comparison to the passive control group, but not to the active control group. While the first observation was probably due to differences in power, the unspecific enhancement in the interference list of Stroop 3 suggests that relaxation training, which was used in the present study as active control and was supposed not to involve cognitive resources, may reduce distractibility (Yesavage and Jacob, 1984). Further research may shed more light on the relationship between relaxation and interference processing.

Our study also aimed at transfer effects. Near transfer indicates performance enhancement in structurally similar tasks involving overlapping neural circuits, whereas far transfer denotes effects on structurally dissimilar tasks (Karbach and Kray, 2009; Karbach and Verhaeghen, 2014; Strobach and Huestegge, 2017). Given that the CT in the present study used games and exercises that clearly differed from the psychometric tests used to evaluate the training effects, we assume to observe some far-transfer effects. It is important to stress the point that the CT did not include any explicit elements of the psychometric tests used for the pre- and post-measures (see the Appendix for a detailed description of the exercises). Thus, increases in post-test relative to pre-test measures of performance would suggest far transfer of training-related gains to specific cognitive functions measured by the tests. This assumption was also supported by the medium to large effect sizes in performance differences between pre- and post-tests after CT. Differences in effect sizes between tests suggest that performance in a test assessing a particular cognitive function benefited more from the training than performance assessed by another test. Therefore, we assume that attentional functions benefited more from CT than crystallized cognitive functions.

However, it has to be noted that near and far transfers have been differently defined in the literature. Some authors proposed that far transfer is given when dissimilar tasks were used for training and evaluating CT effects. For example, Karbach and Kray (2009) used task switching training and evaluated far transfer in a Stroop task that may share the same functional components (e.g., inhibition). Other authors proposed that far transfer occurs when no functional overlaps are shared, for example, when CT improved daily functions in individuals following a training of reasoning (Willis et al., 2006). Barnett and Ceci (2002) proposed a systematic taxonomy of near and far transfers, based on the contextual similarity and dissimilarity of training and test. A clear evidence of (very) far transfer in our study would be a positive effect of multidomain CT on performance in daily activities, as assessed by the CFQ that evaluated attentional and memory lapses in daily life. However, consistent with previous results using CFQ (Strobach and Huestegge, 2017), we did not find group differences, demonstrating similar absent-mindedness and slips of action in all groups of participants. This suggests no far transfer of our CT to daily activities at least in the cognitive functions measured by CFQ.

Nevertheless, as outlined above, far transfer to dissimilar cognitive tasks was found in the present study. This is in line with behavioral and electrophysiological findings obtained in the same study and reported previously, including computer-based versions of task switching (Gajewski and Falkenstein, 2012), Stroop task (Küper et al., 2017), *n*-back task (Gajewski and Falkenstein, 2018), and visual search task (Wild-Wall et al., 2012). All these tasks represent different executive functions but presumably share some characteristics involved in all these tasks. This could be reflected by attentional processes necessary to select particular stimuli but also by cognitive control of action required for successful response selection and execution. There are also indications that CT leads to cognitive improvements in terms of lower within-subject variability in performance (higher consistency) in specific executive processes like response selection that may be supported by attentional gating (Gajewski and Falkenstein, 2012; Gajewski et al., 2017; Küper et al., 2017). In other words, CT may improve selection of task-relevant stimuli and the corresponding activation of stimulus–response mapping, which is critical for successful task performance. This, in turn, may rely on more efficient synaptic transmission or denser neuronal network supporting these functions.

Overall, transfer effects are controversially discussed as the results are rather inconsistent (Melby-Lervåg et al., 2016). It seems that the amount of transfer as well as the efficacy of CT is determined by the type of training (e.g., its total duration and intensity), the participants’ baseline performance, whether or not the training is supervised (Lampit et al., 2014), and probably most important, the intrinsic motivation of the participants (Jaeggi et al., 2014; von Bastian and Oberauer, 2014). Additionally, differences in the efficacy and transfer of CT may be due methodological heterogeneity and structural differences between studies, depending on the trained cognitive domains. A further crucial factor for inconsistency of results is related to the measures used to evaluate training gains like standardized psychometric tests, computer-based tasks, and their specific parameters that often vary between different studies. From this perspective, it would be useful to determine characteristics and to establish guidelines for an optimal combination of factors to enhance the efficacy of CT based on a review of meta-analyses (cf. Bavelier and Green, 2019). For example, a meta-analysis by Lampit et al. (2014) suggests a group-based, multidomain training with session length between 30 and 60 min and frequency of two to three sessions per week to be most effective. The design of the present study met most of these criteria. In addition, regarding the crucial measures, it appears important to use sensitive tasks or psychometric tests with a wide range of possible outcomes to avoid ceiling effects and to ensure that possible improvements by CT can be detected reliably.

### Limitations

There are some limitations of the study that have to be acknowledged. Firstly, the functional overlap between the training and test tasks is not easy to evaluate because of a variety of different games and subtasks that were used. Additionally, paper-and-pencil trainings that trained working memory and speed of processing as well logical thinking were used. The goal of our multidomain training was to maximally enhance cognitive functioning in older adults by offering them an interesting and varying program with a large fun factor to fill more than 30 training sessions and to maintain their motivation to train across this relatively long time.

Secondly, it was not possible to evaluate individual training data to assess interindividual differences in the progress of training-induced gains. This was due to partly using a number of freely available internet games that did not record or permanently store the training data. Moreover, the training difficulty was individually adjusted to the training progress of the participants who absolved individually different training units, making the analysis and interpretation less meaningful.

## Conclusion

The present study provides further evidence for positive effects of trainer-guided multidomain CT on fluid intelligence in older age. In particular, a 16-week-long CT substantially improved attentional endurance and interference processing. These effects were specifically evident for tests that require verbal fluency or selection of predefined targets and ignoring distractors, indicating enhancement of executive functioning like inhibitory control. Moreover, a transfer effect was observed to non-explicitly trained functions, but not to performance in daily life activities. The study contributes to the literature showing positive effects of CT in old age, especially on executive functions that reflect a crucial aspect of cognitive performance highly susceptible to aging.

## Data Availability Statement

The raw data supporting the conclusions of this article can be provided by the authors on request.

## Ethics Statement

The studies involving human participants were reviewed and approved by Ethical Committee of the Leibniz Research Centre for Working Environment and Human Factors, Dortmund. The patients/participants provided their written informed consent to participate in this study.

## Author Contributions

PG designed the study, analyzed the data, and wrote the manuscript. MF designed the study. ST, EW, and SG wrote and revised the manuscript. All authors approved the final version of the manuscript.

## Conflict of Interest

MF was employed by the company Institut für Arbeiten Lernen Altern GmbH. The remaining authors declare that the research was conducted in the absence of any commercial or financial relationships that could be construed as a potential conflict of interest.

## Footnotes

1 www.mentaga.de; www.ahano.de; www.mental-aktiv.de; www.freshminder.de

## Appendix

The appendix contains a detailed description of the exercises included in the cognitive training and the schedule of the cognitive training procedures.

**MAT** (www.gfg-online.de) is a paper and pencil package with short exercises which had to be applied for 10 min daily to increase working memory capacity, visual attention and speed of information processing. In particular, the training aimed at enhancing psychomotor processing by faster perceiving and responding to objects or words, for example detection of triangles in a complex geometric figure or identification of words in a complex letter matrix, which were arranged either vertically, horizontally or diagonally. Memory training included exercises that used words, figures or digits. Participants were asked to memorize the items from each category and recall as many items as possible after several minutes. A more complex exercise consisted of association between faces and personal data like age and profession and recalling the information after a face presentation 10 min later.

The training begins with easy exercises to make quick effects possible. By creating more challenging instructions and by allowing less time for task performance, the level of difficulty gets enhanced gradually.

The training consists of the following modules:

Information processing speed: Time limited visual search. Different forms, numbers and letters are used. Identification of single words in randomly assembled sequences of letters. The hidden words are arranged forwards, backwards, vertically, horizontally or diagonally.

Memory span: Keep several numbers, words or pictures in memory and immediate recall of words or identifying missing words.

Basic learning speed: Memorization of faces with personal data and memorization of faces with distracting stimuli.

**Mental-aktiv** (www.mental-aktiv.de) is an internet based platform that offers a number of memory tasks using digits, letters, colors and figures and exercises to train speed of processing. The exercises were designed in cooperation with the authors of MAT and trained the same functions as listed above.

**Sudoku** is a logic-based number placement puzzle that consists from a 9 × 9 grid with digits so that each column, each row, and each of the nine 3 × 3 sub-grids contain all of the digits from 1 to 9.

**Ahano peds** (www.ahano.de) consists of units with different levels of difficulty. The free available program includes an eye-hand coordination task, money counting task, detection of word repetitions in a text, block taping task, memory for abstract figures etc.

Double:

There is a yellow ball and a red box presented on the screen. With one hand, the participant has to use the computer mouse in a certain way in order to put the ball into the box. With the other hand, the participant has to type the presented words as quickly as possible. This exercise trains peripheral visual attention as well as the coordination of multiple operations.

Euro Coins:

There are many different coins in a purse. The task is to assemble specific coins in order to reach a given amount. This should be done as often as possible within a specific interval. Visual perception, selective attention and mental arithmetic are trained.

Response:

Balloons float past the window of an aircraft. The task is to click as quickly as possible on the relevant balloon appearing on the left side of the window. This exercise trains selective attention and distractor inhibition.

Palpation:

At the time when a green light appears on the screen, one of five given forms is hidden behind a big picture. The participant’s task is to touch the form by use of the computer mouse in order to decide which form is hidden in the current trial. To make a choice, the participant has to click on the corresponding picture. There is only one attempt in each trial. This exercise trains perception and spatial-visual memory.

Double Words:

A pool of words is given, which contains each single word twice. The task is to click on the currently relevant word by use of the computer mouse. There are five attempts in each trial to find the correct word. This exercise trains the participant’s memory.

Chimpanzee test:

Nine fields are presented containing single digits for a short time. After the digit’s disappearance, the participants are instructed to click on the fields in ascending order to reproduce the positions, where the respective figures were shown. Here, visual perception, short-term memory and spatial-visual memory can be trained.

Colors:

The participants have to memorize the colors of a presented picture. The task is to “repaint” the image by first clicking on a “paint pot” and then clicking on the image area. The participants receive one point for each correctly chosen color. Visual perception, short-term memory and spatial-visual memory can be trained by this exercise.

**Mentaga** (www.mentaga.com) consists of exercises enhancing vigilance, perceptual speed, spatial attention etc. like comparison of visual patterns, face learning, counting, vigilance and eye-hand coordination.

Figurative Thinking:

In each trial, two, almost identical pictures are presented. There are exactly three differences between the two pictures, which the participant has to detect as quickly as possible. This exercise is designed to support selective attention.

Capacity:

The task is to catch vertically falling balls with a basket as accurately and quickly as possible. To adjust the basket, the participant has to use the computer mouse. Simultaneously, as many numerical and alphabetical tasks as possible have to be performed. Spatial-visual attention, arithmetic, concentration and of multiple task performance should be improved by this task.

Concentration:

In each trial, an “E” surrounded by a certain number of dots is presented. The task is to identify every E which is surrounded exactly by three dots as quickly as possible. Concentration and visual attention are trained by this task.

Pattern Matching:

Four pictures are presented in each trial. There is always one original, two rotated versions of the original and one differing picture, which the participant has to identify by clicking on it. This exercise trains the abilities of mental rotation and visual search.

Person Memory:

This exercise aims at memorizing and recognizing names and faces. First, a sequence of faces and names is presented and the participants explicitly have to memorize the names. Then, faces are displayed with various names. The participant has to decide which name is related to a particular face. This exercise specifically trains object recognition.

Visual Acuity:

In each trial, two pictures are presented. As quickly as possible, the participant has to decide whether the two pictures are identical. Visual acuity and visual search are trained by this task.

Response Capacity:

Two objects are presented side by side. The participant has to decide whether the objects are identical. A response is required if the objects are identical. This exercise aims at improving visual search and decision time.

Memory for Numbers:

The participant has to memorize and reproduce numbers presented on the screen. The length of each number is adapted to the participant’s capacity. The more digits a number contains, the more time is granted for memorizing and reproducing the number. Primarily, this exercise trains the memory for numbers, but also working memory in general.

**TABLE A1:** A schedule of the cognitive training program.

Week	Session	Exercise
1	1	MAT
	2	MAT
2	3	MAT
	4	MAT
3	5	MAT
	6	MAT/Sudoku
4	7	MAT/Sudoku
	8	MAT/Sudoku
5	9	Mental-Aktiv/Ahano/Sudoku
	10	Mental-Aktiv/Ahano
6	11	Mental-Aktiv/Ahano
	12	Mentaga/Mental-Aktiv/Ahano
7	13	Mentaga/Mental-Aktiv/Ahano
	14	Mentaga/Ahano
8	15	Mentaga/Ahano
	16	Mentaga/Ahano/Sudoku
9	17	Mentaga/Ahano/Sudoku
	18	Mentaga/Sudoku
10	19	Mentaga/Sudoku
	20	Mentaga/Sudoku
11	21	Mentaga
	22	Mentaga
12	23	Mentaga/Ahano
	24	Mentaga/Ahano
13	25	Mentaga/Sudoku
	26	Mentaga/Ahano
14	27	Mentaga/Ahano
	28	Mentaga/Ahano
15	29	Mentaga/Ahano
	30	Mentaga
16	31	Mentaga/Ahano/Sudoku
	32	Mentaga/Sudoku
